# Evaluation of anesthetic skills acquisition in pre-graduate veterinary students with different grades of anesthetic experience using veterinary simulation exercises

**DOI:** 10.3389/fvets.2023.1254930

**Published:** 2023-12-21

**Authors:** Fernando N. Amitrano, Lorenzo E. Quiroz, Ilona R. Jaffe, Nellie G. Goetz, Haverley A. Coy, Robert D. Keegan

**Affiliations:** Department of Veterinary Anesthesia and Surgery, College of Veterinary Medicine, University of Arizona, Tucson, AZ, United States

**Keywords:** anesthesia, education, simulation, veterinary, training

## Abstract

**Background:**

Anesthetic skills are usually learned through continuous supervision by experienced trainers who observe, advise and challenge students. Current educational techniques rely less on live animal training and include the use of simulations and models for teaching and assessment of surgical and anesthetic skills.

**Objective:**

To evaluate the development of anesthetic skills of veterinary students having different levels of previous experience using simulation. An additional aim was to evaluate the impact of the simulation training on students with no anesthesia experience.

**Study design:**

Single group periinterventional and postinterventional study.

**Methods:**

Initial and final anesthesia simulation training recording were obtained from 53 randomly selected veterinary students. Seven faculty members blinded to previous student anesthesia experience reviewed the simulation recording and scored student performance using a rubric, results were recorded and analyzed.

**Results:**

All students participating in an anesthesia and surgery course reached higher proficiency levels on fundamental anesthesia skills regardless of their previous amount of experience with anesthesia. Simulation based learning positively influenced the final score in veterinary students having no previous anesthesia training, suggesting that it is possible for veterinary students to achieve a level of competence in anesthesia skills with simulation-based training.

**Main limitations:**

Sample size, group simulation, multiple reviewers bias.

**Conclusion:**

Students having no experience with clinical anesthesia demonstrated remarkable improvement in their skills, achieving a score that was similar to students having extensive prior clinical anesthesia experience. Despite this clear improvement students having no prior clinical anesthesia experience required more time to complete all anesthesia tasks and may require more training sessions to acquire the speed demonstrated by peers who had significant prior clinical anesthesia experience. Overall, all participants reached a higher proficiency level performing fundamental anesthesia skills at the end of the course.

## 1 Introduction

Of the medical subspecialties, anesthesiology has been recognized as a pioneer in the development of simulation scenarios and training programs for medical students and residents for over 40 years (1, 2), which has led to the development increasingly sophisticated and advanced training simulators. Multiple disciplines in the healthcare profession have recognized the potential benefits of simulation technology for skills training (3). Although simulations are not identical to real life situations, they are able to stimulate real life scenarios and provide instant feedback about decisions, trouble shooting and consequences of actions (2). Previous studies have recognized the improvement in knowledge acquisition, confidence, skills and behaviors in students and health professionals around the world (4, 5). The retention of knowledge when students and residents practice under simulation environments in comparison with traditional lectures has been recognized for many years (6). Students and clinicians trained using simulation have been shown to respond faster, deviate less from accepted guidelines, and perform better when handling intense anesthetic crises (7).

Graduate veterinarians are allowed to perform anesthesia and surgery without any additional training after completing veterinary school making it paramount for the educational institution to provide sufficient clinical training despite a reduction in the use of live animals for teaching (8). Accordingly, veterinary training programs around the world are being equipped with simulation centers having the main objective to train students, interns, and future clinicians (9). Currently, veterinary educators recognize the challenge of teaching pre-clinical veterinary students with the necessary skills and knowledge to fit the requirements of veterinary medicine practice (9). All anesthesia simulation training has the primary objective of improving anesthetic performance and reducing the incidence of critical errors occurring during live animal anesthesia (10, 11).

The goal of our study was to provide evidence for the value of anesthetic simulation training in veterinary students who are learning to manage anesthesia of live animals. An additional objective was to identify anesthesia tasks that create major challenges for veterinary students. Our hypothesis was that veterinary students with no anesthesia experience could become proficient in anesthesia using a program of simulation training.

## 2 Materials and methods

This study was conducted at the simulation operating room suites at the University of Arizona Campus Agricultural Center. Simulation facilities were composed of two operating rooms equipped with advanced computer-generated multiparameter monitor screens. Simulation software (Stage III Simulation Release 2, Academic, WholeLogic, Inc.) was used to generate and display physiologic parameters ECG (electrocardiogram), non-invasive blood pressure, pulse oximetry, capnography, body temperature, oxygen flow rate, anesthetic vaporizer percentage and depth of anesthesia as represented by a palpebral reflex. A commercially available canine training mannequin (Anastasia Canine Mannequin, WholeLogic, Inc.) interfaced with the Stage III software and was used as the canine patient simulator (CPS). Other elements in the simulated operating suite included two identical functional anesthetic machines with a rebreathing circuit, a 2-liter reservoir bag, an oxygen E cylinder with functional oxygen flow to the anesthesia machine, a fully stocked crash cart with appropriately labeled simulated drugs, a blood pressure cuff, gauze sponges, simulated fluids with working fluid lines and suction canister. Six simulation exercises were developed through a standardized, multistage process to effectively simulate and recreate a specific operating room environment. The scenarios included hypotension, bradycardia, pain, CPR and two unknown scenarios. All students underwent a final individual anesthesia and surgery assessment after they had completed all the simulation scenarios to evaluate the skills and clinical development at the end of the course.

The group of veterinary students from the University of Arizona College of Veterinary Medicine class of 2024 that enrolled in Anesthesia and Surgery VETM 814-A: principles of anesthesia and surgery during Spring 2023 were selected for the study. Students enrolled in this course were provided with specific learning objectives and learning material concerning each simulation event. Before each simulation scenario, students were provided with the case clinical information, including patient history and physical exam, surgery to be performed, and drugs chosen for the anesthetic protocol. Also, students were provided with a 30 min recorded anesthesia review lectures as prework for simulation scenarios. All students enrolled in VETM 814-A were randomly preassigned into simulations groups of four students before the course starting date. Two students acted as anesthetist and two as surgeons for each simulation scenario. At the end of the course, each student completed 3 anesthetic simulation training sessions. All simulations were recorded using a recording software system (VALT Video recording and streaming software, Intelligent Video Solutions, Inc.). Immediate feedback was provided during simulation, a faculty expert was located inside an adjacent control room with the main role of adjusting the physiologic states in accordance with predetermined simulation scenarios. During the simulations students were responsible for completing several anesthesia tasks including pressure checking the anesthesia machine, connecting ECG leads: placing the capnograph sampling line, pulse oximeter, non-invasive main blood pressure cuff, temperature probe, esophageal stethoscope, and connecting the intravenous fluids at a proper fluid rate.

A student survey was conducted at the completion of VETM 814-A course, to assess previous anesthesia experience. Anesthesia experience was defined as being responsible for providing general anesthesia to live patients under supervision for longer than 6 months. Although 6 simulation scenarios were provided during the course, each student participated as an anesthetist in three simulation scenarios and participated as a surgeon in the remaining scenarios.

### 2.1 Statistical methods

Of the 110 students enrolled in the course, a total of 106 answered the anesthesia questionnaire survey. Of the 106 students that answered the questionnaire, 52 responded that they had no previous anesthesia experience, 19 responded that they had less than a year of experience and 35 had more than a year of experience. Fifty-four students were randomly selected for the study and, depending upon their experience, they were assigned into the three experience level groups: no experience (NONE), less than a year experience (SOME) and more than a year experience (HIGH). A total of 18 students were included in each group to obtain a 95% confident level from the 19 less than a year experience students samples size. The no experience and more than a year experience group were equal the number as the less than a year experience group. Initial and final anesthetic simulation recordings were obtained from each of the selected students. Seven faculty members, blinded as to student level of experience, reviewed the same student initial and final simulation recordings, and scored the student’s anesthetic skills performance using a rubric (Table 1). Rubric scores were divided into four categories: constant guidance (score 1), intermittent guidance (score 2), on demand guidance (score 3) and no guidance (score 4). For training purposes and to minimize the stress level of students, all initial simulation scenarios were performed in pairs except for their final simulation assessment. For this reason, a not performed category was included in the initial anesthesia rubric in case the evaluated student did not perform the anesthesia task listed in the rubric. Each of the students was individually scored by one faculty member, however 21 students selected for the trial were randomly reassigned to be evaluated by the second faculty member to improve the score reproducibility. If a disagreement occurred during the grading period, the main author reviewed the video and decided the final score.

**Table 1:** Anesthesia rubric.

Anesthesia skills/grades	Constant guidance	Intermittent guidance	On-demand guidance	No guidance required	Not performed
Correctly pressure check the anesthesia machine					
Properly connect ECG leads (using ECG gel)					
Correctly placed airway sampling line (EtCO2 capnograph line)					
Correctly placed the non-invasive main blood pressure cuff					
Correctly placed Pulse Ox probe					
Correctly placed temperature probe					
Connect and deliver IV fluids at proper rate					
Correctly placed esophageal stethoscope					

Rubric scores categories: constant guidance: score 1, intermittent guidance: score 2, on demand guidance: score 3 and no guidance score 4.

A mixed statistical analysis using ANOVA was used to evaluate the average performance between the initial assessment and the final assessment for students with different levels of experience. Individual anesthetic tasks and time of task completion were evaluated using a Wilcoxon signed rank test, and finally we analyzed the variation in task completion time associated with the previous anesthesia experience level of the participating student partner during the pre-course simulation using a mixed model analysis of ANOVA. Level of significance was set using an alpha level of 0.05.

## 3 Results

### 3.1 Students

A total of 110 veterinary students enrolled in the spring 2023 VETM 814-A course at University of Arizona College of Veterinary Medicine. Of the students enrolled in this course, a total of 106 answered the anesthesia questionnaire survey. Eighteen students were randomly selected for each experience level group.

### 3.2 Initial assessment

As expected, the initial assessment total performance mean task score was significantly higher at 3.6 (95% CI 3.4–3.8) for experienced students in comparison with non-experienced students (mean score of 2.9, 95% CI 2.7–3.1) (Figure 2). Pressure-checking the anesthesia machine was the most difficult task for all the participants with a mean score of 2.09 (95% CI1-3) (Table 2). The total time for completion of all the anesthesia tasks was 7:03 min and it was significantly lower for students having more than 1 year of experience with a mean time of 6:23 min versus 7:02 min SOME and 7:40 min NONE (Figure 2). Mean task completion times in the pre course evaluation were significantly lower only when student pairs were comprised of students having disparate amounts of clinical experience. Student pairs having similar pre course experience levels took longer to complete the tasks (7:58 min) compared with pairs comprised of an experienced and inexperienced student (Figure 2).

**Figure 1:**
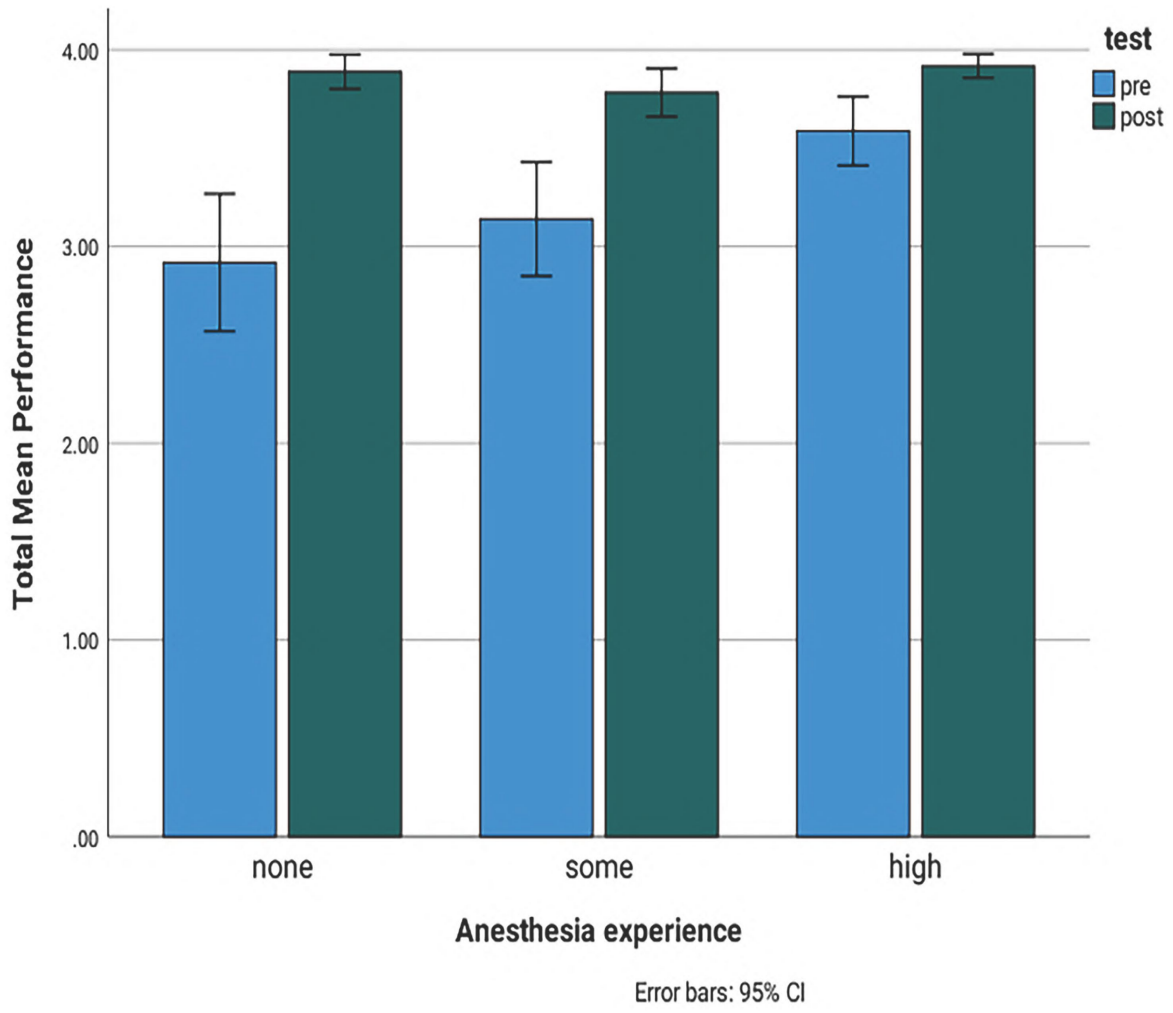
Bar chart showing the comparison of the total initial and final assessment mean performance scores for students with no experience, less than a year experience and more than a year experience. Pre: initial assessment. Post: final assessment. None: no experience. Some: less than a year experience. High: more than a year experience. 95% CI: 95% confidence interval light blue bar: initial assessment scores. Dark green bar: final assessment scores. Score 1: constant guidance, Score 2: intermittent guidance, Score 3: on demand guidance and Score 4: no guidance.

**Table 2:** Total median of differences between initial and final assessment mean scores for students performing anesthesia task under simulation.

Anesthesia task	Total score initial assessment (*n* = 54)	Total score final assessment (*n* = 54)
Correctly pressure check the anesthesia machine	2.09	3.70
Properly Connect ECG Leads	3.45	3.96
Correctly place airway sampling line (EtCO2 capnograph line)	3.38	3.93
Correctly placed the non-invasive main blood pressure cuff	3.60	3.85
Correctly place pulse oximetry probe	3.78	3.98
Correctly place temperature probe	3.07	3.91
Connect and deliver IV fluids at proper rate	3.29	3.85
Correctly place esophageal stethoscope	3.05	3.45

### 3.3 Final assessment

**Figure 2:**
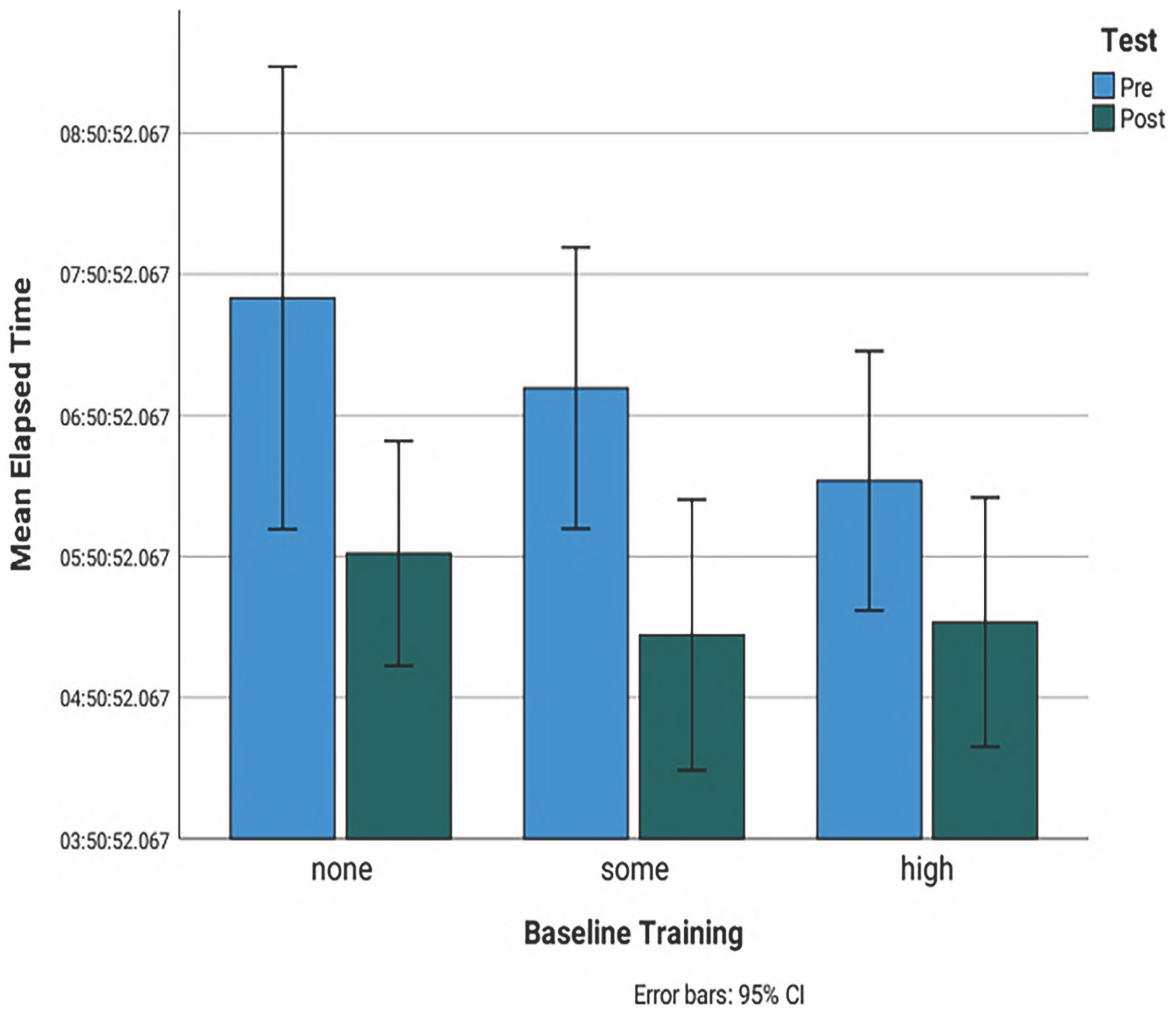
Bar chart showing the comparison of the total mean time of initial and final assessment for students with no experience, less than a year experience and more than a year experience. Pre: initial assessment. Post: final assessment. None: no experience. Some: less than a year experience. High: more than a year experience. 95% CI: 95% confidence interval light blue bar: initial assessment scores. Dark green bar: final assessment scores. Score 1: constant guidance, Score 2: intermittent guidance, Score 3: on demand guidance and Score 4: no guidance.

On the final individual simulation assessment there was a significant increase in the mean task scores for students having no prior experience (NONE) with a mean score of 3.78 versus SOME 3.88 and HIGH 3.92 (Figure 2). Following simulation training, time of completion was significantly reduced in all groups with a total mean time of 5:30 min in comparison with their individual pre simulation training assessment. Despite the general time improvement, the non-experienced group of students still required slightly more time than the two experienced student groups to accomplish all the anesthetic tasks with a mean time of 5:52 min versus SOME 5:17 and HIGH 5:22 (Figure 2).

### 3.4 Reviewers

A total of 21 students were evaluated by two faculty members accounting for 39% of the total examined participant. There was moderate viewer agreement (70%) from a total of 291 evaluated tasks. The two categories with highest disagreement were on demand and no guidance (41%), with the placement of the ECG leads task causing the most number of disagreement between reviewers (17%).

## 4 Discussion

Previous reports showed that students and health professionals trained using a high-fidelity anesthesia simulation responded to an event more rapidly, performed better and were more willing to accept constructive criticism compared with students that were trained using routine didactic methods (1, 2, 5, 7). In this study we reported the results of the ability of veterinary students to acquire and improve anesthetic skills after simulation training. These findings are important to veterinary education and support the use of immersive anesthesia simulation as an effective teaching tool. To our knowledge there are no prior studies in veterinary medicine that report the interaction between prior anesthesia clinical experience and simulation training in veterinary students. Our work provides evidence that students with little to no experience can train using immersive simulation and reach a performance level equal to students having high previous clinical anesthesia experience. The final assessment scores from students having no prior clinical anesthesia experience were higher in comparison with their initial assessment scores demonstrating the effectiveness of the simulation training program. In addition, completion time was significantly reduced showing the increase of effectiveness and speed acquired by students after simulation.

The anesthesia and surgery course developed and implemented by our institution is a novel approach to training and integrates both anesthesia and surgery. Anesthesia is both stressful and demanding making the attainment of skill and knowledge critically important (10). Multimodal training courses in veterinary anesthesia employing simulation training with live animal experiences have been shown to provide a significant increase in student knowledge and self-efficacy (8). Trainees can make mistakes and learn to recognize and correct them during simulations, minimizing fear of harming actual patients while delivering high quality knowledge and professional skills (3, 8). Simulation training can prevent mistakes and deliver a better patient care experience (2, 3) An analogous course of technical and cognitive skills training has been used in surgical instruction for many years (12, 13). These programs are designed to train and evaluate individuals from a broad range of surgical specialties on their knowledge and technical skill (13), providing a standard technical proficiency prior to performing a surgical procedure. These same fundamentals are applied to all the students that enrolled in the VETM 814-A course at the University of Arizona. At the end of the course an individual assessment is conducted, and all students are objectively evaluated to demonstrate the ability to perform anesthesia on a live patient.

The authors recognize the potential limitation of the study. To minimize the stress level during simulation it was decided that students were placed in groups of two during all their training scenarios. Initial assessments were observed on a pair of students performing one set of procedures. For this reason, some tasks could not be fully evaluated by the reviewers and may have contributed to the disagreement in grading of the initial recordings due to only one member of the pair performing the task or when a more experienced student instructed the less experienced student thus creating the impression of lack of skills. In our opinion, this peer instruction is beneficial in the development of skills as students learn from their more experienced peers. However, it looks like paring students with a similar level of training has no benefit. This might be related to students having the same knowledge gaps minimizing peer-to-peer learning. From our knowledge, we believe that students with no experience obtained direct dynamic training example and directions, creating a positive learning atmosphere and experience, minimizing the fear of being corrected by the facilitator and gradually obtaining more confidence on their skills performance.

A well-known limitation of simulation training, time restriction, was encountered in our study. Simulation time is necessarily restricted and consequently much of the learning during a simulation session is dependent upon individual student engagement and teamwork during the session (10). As a consequence, the final time required by students having no prior anesthesia experience to complete their anesthesia tasks was significantly higher compared with students having prior clinical anesthesia experience. This is not an unexpected finding and further studies to assess the number of simulation sessions required to match the level of pre simulation clinical experience are warranted.

Results of this study demonstrate that we were able to train veterinary students with no previous experience on basic anesthesia tasks using three immersive simulation training sessions. Indeed, all non-experienced participants reached an acceptable level of proficiency after three immersive simulation sessions. Opportunity exists for further research into the design and implementation of immersive veterinary anesthesia scenarios.

## 5 Conclusion

Simulation continues to be an exceptional way to train students. This study provides additional information regarding immersive simulation training and supports the results of prior studies that demonstrate how implementation of multimodal anesthesia training into veterinary medicine curriculums can improve student preparation. Students participating in this study acquired a higher level of proficiency regardless of their previous clinical experience. Providing students with three immersive simulation scenarios was sufficient to increase their basic skill level. Students having no experience with clinical anesthesia prior to the course demonstrated remarkable improvement in their skills, achieving a score that was similar to students having extensive prior clinical anesthesia experience. Despite this clear improvement students having no prior clinical anesthesia experience required more time to complete all anesthesia tasks and may require more training sessions to acquire the speed demonstrated by peers who had significant prior clinical anesthesia experience. Overall, all participants reached a higher proficiency level performing fundamental anesthesia skills at the end of the course in comparison with their initial anesthesia course assessment. In addition with the previous mentioned significant advantages of using simulation training, we strongly believe is a non restrictive educational because it does not require any experience level or special skills to submerge students into live like scenarios. Also, experience students can learn new techniques or increase their efficiency and speed of the already acquired ones previous to simulation training.

In summary, there is a clear need for further expansion of anesthesia skills/simulation training in veterinary education to offer a safe and feasible option to traditional, live animal-based training approaches. To meet current educational challenges, veterinary teaching programs must continue to investigate the value of simulation training and incorporate this practice into their curriculum.

## Data availability statement

The original contributions presented in the study are included in the article/supplementary material, further inquiries can be directed to the corresponding author/s.

## Author contributions

FA: Conceptualization, Data curation, Formal analysis, Investigation, Methodology, Project administration, Resources, Validation, Visualization, Writing – original draft, Writing – review & editing. LQ: Conceptualization, Data curation, Formal analysis, Investigation, Methodology, Resources, Validation, Visualization, Writing – review & editing. IJ: Conceptualization, Data curation, Formal analysis, Investigation, Writing – review & editing. NG: Conceptualization, Data curation, Formal analysis, Investigation, Writing – review & editing. HC: Conceptualization, Data curation, Formal analysis, Investigation, Writing – review & editing. RK: Conceptualization, Data curation, Formal analysis, Investigation, Writing – review & editing.
